# Comparison of Extracapsular Stabilization Techniques Using an Ultrasonically Implanted Absorbable Bone Anchor (Weldix) after Cranial Cruciate Ligament Rupture in Cats—An In Vitro Study

**DOI:** 10.3390/ani11061695

**Published:** 2021-06-07

**Authors:** Lydia Koch, Barbara Bockstahler, Alexander Tichy, Christian Peham, Eva Schnabl-Feichter

**Affiliations:** 1 Small Animal Surgery, Department for Small Animals and Horses, University of Veterinary Medicine Vienna, 1210 Vienna, Austria; lydia.koch@vetmeduni.ac.at (L.K.); barbara.bockstahler@vetmeduni.ac.at (B.B.); 2 Bioinformatics and Biostatistics Platform, Department of Biomedical Sciences, University of Veterinary Medicine Vienna, 1210 Vienna, Austria; alexander.tichy@vetmeduni.ac.at; 3 Movement Science Group, University Equine Hospital, Department for Small Animals and Horses, University of Veterinary Medicine Vienna, 1210 Vienna, Austria; christian.peham@vetmeduni.ac.at

**Keywords:** cranial cruciate ligament rupture, cat, fabellotibial suture, absorbable bone anchor

## Abstract

**Simple Summary:**

One reason for lameness in cats is the rupture of the cranial cruciate ligament. This ligament is located in the stifle joint and contributes to its stabilization during excessive forward movement and internal rotation of the tibia. One method for the surgical treatment of cranial ligament rupture is the placement of an extracapsular suture. Different materials and methods of suture fixation have been used in dogs and cats. This study investigated the use of a novel polylactide absorbable bone anchor that was implanted with ultrasound technology for suture fixation and compared this with suture fixation alone and fixation with a nonabsorbable bone anchor using an ex vivo modified limb-press model. For evaluation, distance measurements on radiographs were performed and the angles between defined bony structures were calculated. The acquired measurements accounted for both craniocaudal and mediolateral movements, and the results showed that the absorbable anchor could neutralize excessive movement within the stifle joint in two of three measurements and seems to be a good alternative to well-known surgical methods.

**Abstract:**

Background: This study evaluated joint stability after surgical repair of cranial cruciate ligament (CrCL)-deficient stifle joints in cats using a novel absorbable polylactide bone anchor in an ex vivo model. Methods: Thirty-six hindlimbs from cats with intact (G_i_ group) and transected CrCLs were treated with fabellotibial suture alone (G_FW_ group), suture combined with an absorbable polylactide bone anchor (G_WD_ group), or suture combined with a nonabsorbable bone anchor (G_FT_ group), positioned in a limb press with predefined joint angles (stifle joint: 120 ± 5°; hock joint: 120 ± 5°) and loaded with 10%, 20%, and 30% of body mass (BM). Predefined points were measured on lateral radiographs and with a coordinate measurement machine. Distances on radiographs (mm) were measured and angles (°) were calculated to represent the craniocaudal movement and the internal rotation of the tibia. Results: There were no differences for craniocaudal movement between G_i_ and G_FW_ or G_FT_, but for G_WD_ regarding angle measurement at 30% BM. For internal rotation, there was no significant difference between G_i_ and G_FW_ or G_WD_, but for G_FT_. Conclusion: The used absorbable polylactide bone-anchor was able to stabilize the stifle joint regarding internal rotation and craniocaudal movement as calculated from distance measurements.

## 1. Introduction

The cranial cruciate ligament (CrCL) is an important anatomical structure in the stifle joint. Its functions include prevention of cranial tibial drawer, excessive internal rotation of the tibia, and hyperextension of the stifle joint [1,2,3]. CrCL disease occurs less commonly in the feline species as opposed to canines and humans [1,3]. Conservative management may seem to resolve the lameness and its associated pain but most of these cats will continue to have some degree of cranial tibial translation [3,4,5,6]. Surgery is thus recommended to prevent continued stifle instability and provide a quicker and more reliable return to function [3,5]. Tibial-plateau-leveling osteotomy (TPLO) and tibial tuberosity advancement (TTA) are commonly used to treat CrCL disease in dogs [7,8,9,10,11,12,13]. TPLO and TTA however has only been investigated in a few studies in cats, whereas extracapsular stabilization with fabellotibial suture has been reported more commonly [5,12,14,15].

Limb-press models have been widely used in research studies to investigate different stabilization methods in dogs and cats [8,10,13,14,16,17,18]. Stability in the stifle in these limb-press models has been evaluated using either radiographic measurements [16,18] or a three-dimensional coordinate-measuring system when comparing different surgical techniques [18].

The use of ultrasonically implanted absorbable bone anchors has been investigated in sheep for treatment of mandibular fractures, spinal fractures, skull reconstruction and for implantation in the femur and tibia [19,20,21,22]. In humans, these implants have been used for tendon and ligament repair and for joint stabilization [23,24]. In cats and dogs, there are no published reports on their clinical use. In contrast the use of conventional bone anchors for suture placement in the femur during extracapsular stabilization has been well described in dogs, and it has been shown to be an effective option for the surgical treatment of CrCL rupture [25,26,27]. However, there are no such studies evaluating the use of bone anchors for fabellotibial suture fixation in cats. In this study we investigated the surgical stabilization of the CrCL-deficient stifle joint in cats, using either an ultrasonically implanted absorbable polylactide bone anchor or a conventional anchor in an ex-vivo model.

The absorbable polylactide anchor is implanted with BoneWelding technology (VetWelding AG, Stansstad, Switzerland), which uses ultrasound for implantation [21,28]. The ultrasound is used to liquefy the anchor polymer at the bone contact interface. The liquid then flows into cancellous bone cavities and immediately solidifies, creating a stable bond with the bone [21,28]. At 37 °C, there is no alteration in the anchor’s pull-out strength for a period of 12 months [29,30]. This period is estimated to be about 6 months for live animals, given that their body temperature is usually higher [29,30]. This time period is also sufficient for the formation of periarticular fibrosis which is ultimately responsible for long-term joint stabilization [29,30]. The benefits of this implantation method include the ability to withstand higher pull-out forces, faster implantation, with no evidence of inflammation at the implantation site compared with the use of conventional bone screws [19,20,21,28,31].

We hypothesized that a fabellotibial suture technique using this ultrasonically implanted absorbable polylactide bone anchor would neutralize excessive craniocaudal drawer and prevent internal rotation of the tibia, after CrCL rupture in cats. We also hypothesized that this technique would be comparable to using a fabellotibial suture either on its own or in combination with a nonabsorbable bone anchor.

## 2. Materials and Methods

### 2.1. Specimens

Hindlimbs (*n* = 36) from cats euthanized for reasons unrelated to the study were harvested from the Hospital for Small Animals of the University of Veterinary Medicine Vienna, Austria. Prior signed consent from the owners was obtained for using the cadavers for teaching or research. The limbs were free from stifle pathology as evaluated by radiography. The recorded body mass (BM) was 3.6 ± 1.1 kg (range 1.7–6.2 kg), and the age was 12.4 ± 5.3 years (range 2.0–19.4 years).

The limbs were prepared as previously described [16,18]. Briefly, they were disembodied, and the soft tissues from the femoral head to the proximal metatarsus were removed. Stifle and talocrural joint capsules, patellar tendon, fabellae, and collateral ligaments were preserved. The femoral head and neck were removed using a saw. The hindlimbs were wrapped in saline-soaked towels and stored at −20 °C until testing.

Prior to testing, the hindlimbs were thawed at room temperature. The femoral shaft was fixed in a copper tube containing polymethyl methacrylate. To simulate the quadriceps mechanism, a wire was guided through a predrilled hole in the center of the patella and connected to a force gauge mounted on the top plate of the limb press. Two 2.0 mm cortical bone screws (Braun, Austria) of 6.0 mm length were horizontally placed in a medio-proximal orientation to both fabellae. A wire was placed around the screws and connected to a turnbuckle which, in turn, was connected to a wire guided through a predrilled hole in the proximal aspect of the calcaneus to simulate the Achilles tendon mechanism.

### 2.2. Limb Press

**Figure 1:**
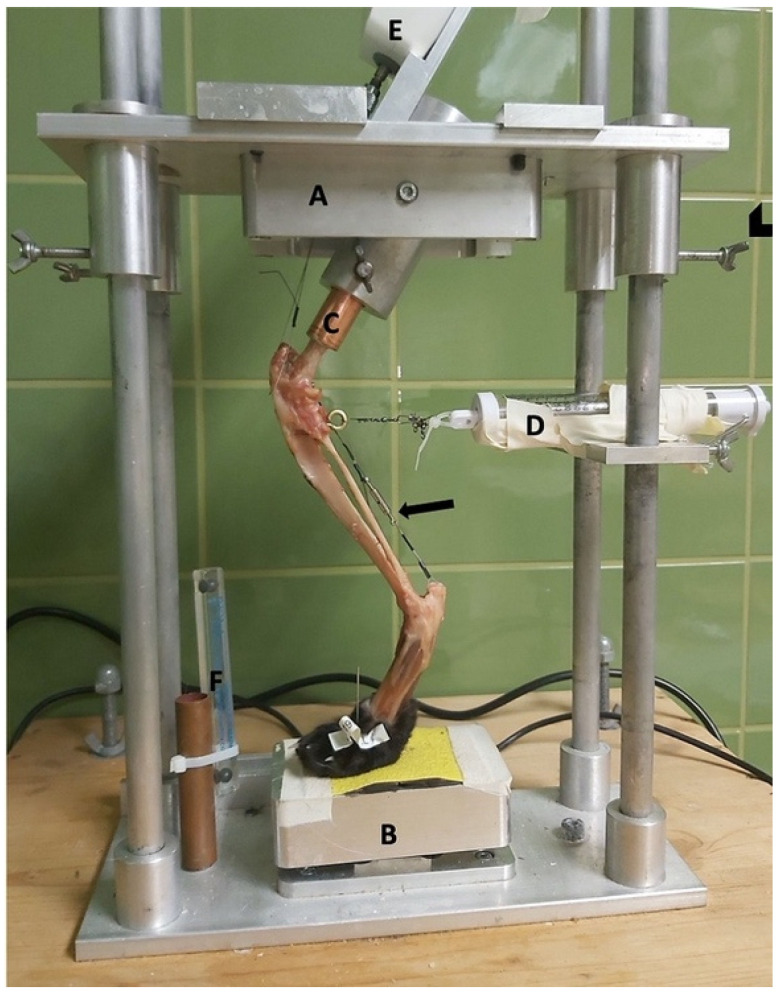
Limb press. A = top plate; B = pedestal; C = copper tube holding the femoral shaft; D = spring mechanism to avoid slack; E = force gauge; and F = radiographic magnification marker (Biomedtrix, Whippany, NJ, USA). Arrowhead = screws to fixate the top plate. Arrow = Achilles tendon mechanism.

For mechanical testing, a limb press was used that has been adapted for feline hindlimbs by Kneifel et al. [16,18]. It comprised a rectangular base plate connected by a column (90° angle) in each corner to an equal-sized top plate. Connection was achieved over drilled holes allowing the top plate to slide up and down and to be secured with screws. To measure the created patellar tendon load (PTL), a device mounted on the top plate held a force gauge (Sauter FA-100; Sauter GmbH, Vienna, Austria) connected to the wire, simulating the quadriceps mechanism. The paw was placed on a pedestal with a rough surface and additionally fixed with a K-wire. The pedestal was placed in the center of a scale (WPT 30F1/K, 0.01 kg digit; Radwag Wagi Elektroniczne, Krakow, Poland) to measure the body mass applied to the hindlimb (Figure 1). In addition, at 0.5 cm distal to the stifle joint surface, the caudal aspect of the tibia was connected to a spring mechanism to keep the tibial plateau from translating cranially relative to the femur.

On the back, a radiographic plate was attached parallel and sagittally to the limb.

### 2.3. Surgical Treatment

For different treatment methods, the hindlimbs were randomly divided into the three groups. For each group, appropriate placement of the created bone tunnels was obtained using radiographs.

#### 2.3.1. Fabellotibial Suture Alone

The first group (G_FW_) underwent a fabellotibial suture technique with #2 braided nonabsorbable multistrand polyethylene suture (FiberWire; Arthrex Vet Systems, Frechen, Germany) without the use of a bone anchor.

The suture was placed through the lateral femorofabellar ligament, guided medially through a bone tunnel cranial to the proximal aspect of the extensor groove of the tibia [32], and guided back laterally under the distal aspect of the patellar tendon and knotted with the opposite end of the suture. Tightening was performed with the mentioned force gauge, at 20 N, as previously described, while the stifle joint was held at an angle of 100° [16,18,33,34] and fixation was performed with six square knots.

#### 2.3.2. Fabellotibial Suture with a Nonabsorbable Suture Anchor

The second group (G_FT_) underwent fabellotibial suture treatment with a 2.8 mm × 11.7 mm threaded nonabsorbable suture anchor (FASTak, Arthrex Vet Systems) preloaded with #2 braided nonabsorbable multistrand polyethylene suture (FiberWire; Arthrex Vet Systems). The hole for anchor placement in the caudal aspect of the lateral femoral condyle was drilled using a 2.0 mm drill bit (Arthrex Vet Systems). Further suture placement and tightening were performed as described for the G_FW_ group.

#### 2.3.3. Fabellotibial Suture with an Absorbable Suture Anchor

The third group (G_WD_) received a 2.3 × 7.2 mm polylactide absorbable bone anchor (Weldix; VetWelding AG) which can be preloaded with different types of suture materials from sizes USP#4-0 to #2. For anchor placement, an ultrasound device (BoneWelder Vet; VetWelding AG) with a frequency of 30 kHz was used.

During implantation, ultrasonic vibrations established shear forces at the anchor–bone interface, which cause the polymer to liquefy and flow into the surrounding cancellous bone structure. The polymer immediately solidifies, creating a stable bond between implant and bone. This is also the case for the used suture material, which is locked in place during this process.

**Figure 2:**
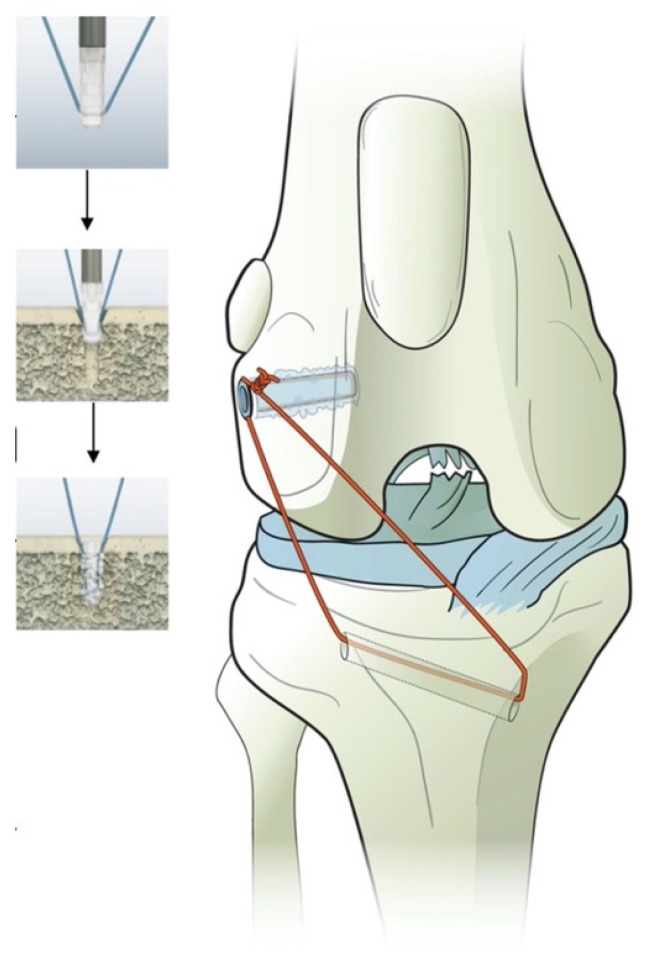
Illustration of the polylactide absorbable bone anchor (Weldix; VetWelding AG, Stansstad, Switzerland), its position in the femoral condyle and the bone tunnel through the tibia. The smaller images show the process of implantation from top to bottom.

In our study, the anchor was preloaded with #2 braided nonabsorbable multistrand polyethylene suture (FiberWire; Arthrex Vet Systems) (Figure 2). A 1.8 mm twist drill with a drill stop (VetWelding AG) was used to create the insertion site in the caudal aspect of the lateral femoral condyle.

Further suture placement and tightening were performed as described for the G_FW_ and G_FT_ groups.

### 2.4. Mechanical Testing

All hindlimbs (n = 36) were tested in the same setup with intact (Gi) and transected (G_T_) CrCL, including after surgical repair (G_FW_, G_FT_, and G_WD_ groups).

**Figure 3:**
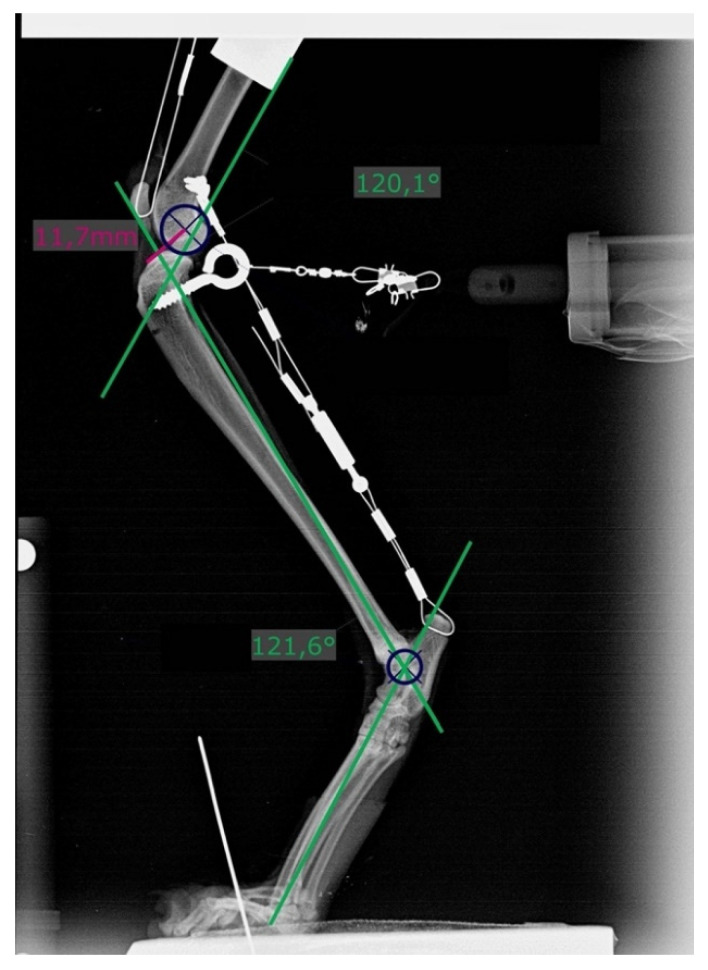
Radiograph showing measurement of stifle and tarsal joint angles (green lines) on the left hindlimb with a transected cranial cruciate ligament and loaded with 20% body mass. Dark blue circles (*r* = 6 mm) are superimposed over the caudal aspect of the femoral condyles. Distances were measured from its center to the most cranial point of the tibial plateau (pink line).

The hindlimb was placed in the limb press, and the paw was secured with a small K-wire. Throughout the experiment, the hindlimb was sprayed with saline to keep it moist. Next, 10%, 20%, and 30% of BM was applied to the hindlimb by sliding the top plate up and down and tightening or loosening the turnbuckle in the Achilles tendon mechanism. The angles of the stifle and talocrural joints did not exceed 120 ± 5° [14,16,18]. Radiographs were obtained for verification (Gierth HF 80/15, X-ray tube; Riesa, Germany) and immediately developed (Kodak Point-of-Care CR-360 System; Carestream Health Inc., Rochester, NY, USA). Distances were measured using the DICOM viewer (dicomPACS view, version 5.2.11; Oehm and Rehbein GmbH, Rostock, Germany), and joint angles were measured as previously described (Figure 3) [14,16,18]. BM and percentage of BM for each cat can be found in the Supplementary Materials (Tables S1–S26).

#### 2.4.1. Patellar Tendon Load

The PTL reflects the tension created within the quadriceps mechanism. It was evaluated thrice at each BM load with an intact CrCL with the value read directly from the force gauge, and the mean was calculated and rounded to the nearest whole number. This value was used for all further testing in both hindlimbs of the same cadaver, guaranteeing the same tension within the quadriceps mechanism, even when altering the top plate or the Achilles tendon mechanism. In cases where the PTL changed when altering the entire mechanism to apply the calculated weights, it was corrected again.

PTL for each cat and each BM load are listed in the Supplementary Materials (Tables S1–S26).

#### 2.4.2. Measurement Procedure

The hindlimb was placed in the limb press, and the described body mass and evaluated PTL were applied. If the joint angles evaluated on the radiographs were within the predefined range, we measured the distance between the most cranial aspect of the medial tibial condyle contributing to the articular surface [35] and the center of the circle (*r* = 6 mm) superimposed over the caudal aspect of the femoral condyles. Subsequently, 3D measurements for later angle calculation were performed using a coordinate-measuring machine (CMM) (Microscribe M; Ravware Inc., Raleigh, NC, USA). The predefined femoral points measured were the proximal aspect of the trochlear groove (F1) and the lateral aspect of the lateral fabella (F2). A point 1 cm distal to the tibial crest (T1) and the fibular head (T2) represented the points on the lower leg. Each point was measured thrice, and the mean was calculated. After first collecting measurements from a hindlimb with an intact cranial CrCL, the cranial CrCL was then transected with a #11 blade, the arthrotomy was closed with interrupted sutures using a 2/0 monofile absorbable suture material (Biosyn; Covidien, Dublin, Ireland), and the hindlimb was, again, placed in the limb press. Previous loads were applied, radiographs were taken, and the coordinates of the predefined points were measured again. After surgical repair, the hindlimb was again mounted on the limb press, radiographs were taken, and the coordinates were measured.

No parts of the limb press were altered when removing a hindlimb, during the time the hindlimb was not in the apparatus, and when replacing the hindlimb.

### 2.5. Calculations

#### 2.5.1. Distances

Two points each on the femur and tibia were defined, and the distance (D) between them was measured on previously obtained radiographs. X1 was the most cranial point of the tibia and X2 the center of a circle (r = 6 mm) superimposed over the caudal aspect of the femoral condyles (Figure 3). If a double condylar sign with a distance exceeding 1 mm was present, the hindlimb was replaced and the measurements performed again.

Differences between the distances measured in the hindlimbs under all conditions, i.e., intact (D_i_) and transected (D_T_) CrCLs and after surgical repair (D_FW_ = fiber wire; D_FT_ = FASTak; D_WD_ = Weldix), were calculated to determine the cranial motion of the tibia under each condition, including D_T_ − D_i_ for assessing the instability gained and D_i_ − D_FW/FT/WD_ for evaluating the remnant instability after each treatment. A difference of 0 indicated the restoration of stability.

Calculated distances for each hindlimb can be found in the Supplementary Materials (Tables S1–S26).

#### 2.5.2. Angles

**Figure 4:**
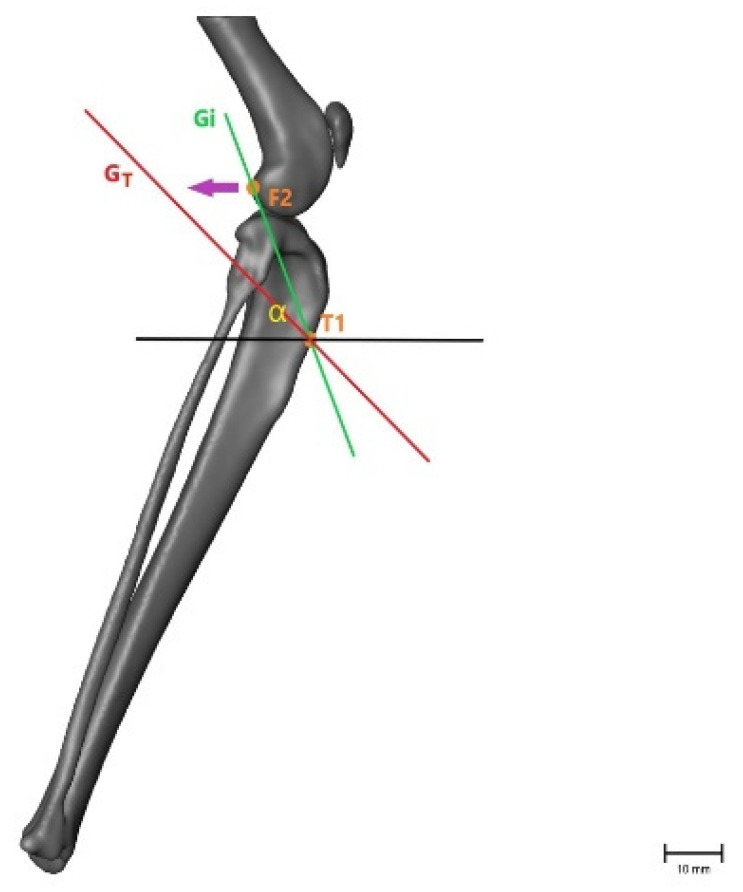
Illustration of the changes within the angle representing craniocaudal movement (α), showing that the angle becomes smaller after CrCL transection as the tibial crest (T1) is cranially displaced. α = measured angle; orange point T1 = point 1 cm distal to the tibial crest; orange point F2 = lateral aspect of lateral fabella in an intact CrCL; green line G_i_ = vector between measured points with an intact CrCL; red line G_T_ = vector between measured points with a transected CrCL; violet arrow = illustration of movement when the CrCL is transected. Scale bar indicates 10 mm.

**Figure 5:**
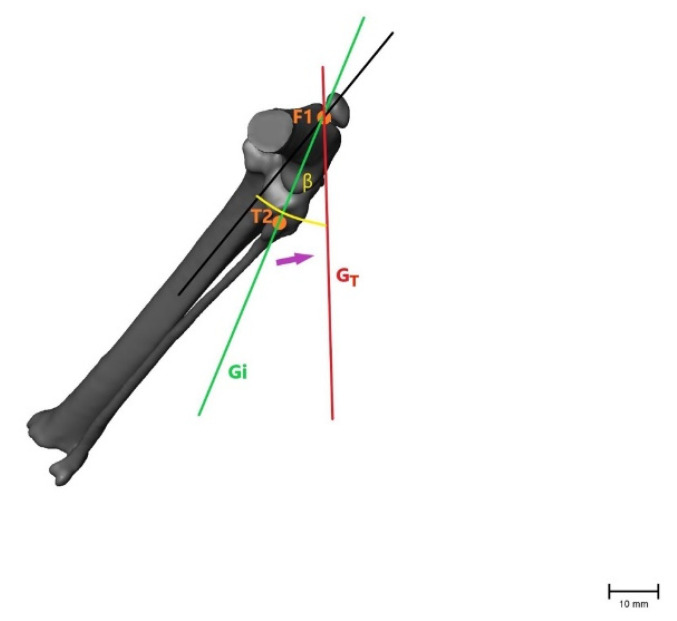
Illustration of the changes within the angle representing internal rotation of the tibia (β), showing that the angle becomes larger after CrCL transection as with internal rotation of the tibia when the fibular head (T2) moves laterally. β = measured angle; orange point T2 = fibular head in an intact CrCL; orange point F1 = proximal aspect of the trochlea groove in an intact CrCL; green line G_i_ = vector between measured points with an intact CrCL; red line G_T_ = vector between measured points with a transected CrCL; violet arrow = illustration of movement when the CrCL is transected. Scale bar indicates 10 mm.

To evaluate the movement between the femur and tibia in more than one plane, the angles between the two measured coordinates were calculated using a 2-argument arctangent. Points F2 and T1 were used to evaluate the craniocaudal movement of the tibia relative to the femur in a sagittal plane, creating angle α. Points F1 and T2 were used to calculate angle β, representing the inward rotation of the tibia in the transversal plane. As the tibia moves forward and rotates internally in the CrCL-deficient stifle joint, angle α decreases and angle β increases (Figure 4 and Figure 5). The angles were calculated for intact (α_i_, β_i_) and transected (α_T_, β_T_) CrCLs and after treatment (α_FW_/β_FW_ = fiber wire; α_FT_/β_FT_ = FASTak; α_WD_/β_WD_ = Weldix). α_T_ − α_i_ and β_T_ − β_i_ were calculated to evaluate the instability gained after transection, and α_i_ − α_FW/FT/WD_ and β_i_ − β_FW/FT/WD_ were calculated to evaluate the remnant instability after each treatment. A difference of 0 indicated the restoration of stability.

Calculated angles for each hindlimb can be found in the Supplementary Materials.

### 2.6. Statistical Analysis

Prior to the commencement of the study, the sample size was estimated using G*Power v3.1 (Heinrich-Heine-Universität Düsseldorf; Düsseldorf, Germany) with a power of 80% to select the adequate number of specimens to be tested. Statistical analyses were performed using IBM SPSS version 19 (IBM; Armonk, NY, USA). To evaluate the correlation between the technique, calculated differences, and load, a general linear model with repeated measurements was used, followed by post hoc tests using Bonferroni’s alpha correction for multiple comparisons.

To evaluate the impact of the technique on the differences for each load, one-sample *t*-tests were used. Pearson’s correlation coefficient was used to detect the correlation between the BM and calculated differences, and the Kolmogorov–Smirnov test was used to determine whether data were normally distributed. *p* < 0.05 was considered to indicate statistically significant differences.

## 3. Results

### 3.1. Distances

The distances measured after CrCL transection were all significantly higher than those in stifle joints with an intact CrCL and increased with higher loads. Following stabilization with braided nonabsorbable multistrand polyethylene suture (FiberWire; Arthrex Vet Systems) (G_FW_ group) and a polylactide absorbable bone anchor (Weldix; VetWelding AG) (G_WD_ group), the measured distances at all loads were not significantly different compared with those of stifle joints with an intact CrCL according to the one-sample *t*-test (Table 1).

The nonabsorbable bone anchor (FASTak; Arthrex Vet Systems) preloaded with #2 braided nonabsorbable multistrand polyethylene suture (FiberWire; Arthrex Vet Systems) (G_FT_ group) showed a statistically significant difference regarding craniocaudal movement compared with the distances measured for an intact CrCL (Table 1). There was a significant effect of load (*F* = 163, *p* < 0.01) and condition (*F* = 545, *p* < 0.01) on the distances, but we could not show an interaction between applied loads and the technique in this setting (F < 1, *p* = 0.71). Pearson’s correlation analysis showed no significance between the BM and distances under all conditions and at all loads.

### 3.2. Angles

Angle α was significantly lower, while angle β was significantly higher in stifle joints with a transected CrCL than in those with an intact CrCL. At a load of 30% BM, there was a significant difference between G_WD_ group and the G_i_ group regarding angle α (*p* = 0.04) (Table 2). The calculated angle β values showed no difference compared to the G_i_ group for all treatments (*p* > 0.05) (Table 3).

The load (F_alpha_ = 10, F_beta_ = 14, *p* < 0.01) and condition (F_alpha_ = 162, F_beta_ = 221, *p* < 0.01) significantly affected angles in the generalized linear model. In addition, the load–condition interaction affected the measured angles (F_alpha_ = 9, F_beta_ = 12, *p* < 0.01). Pearson’s correlation showed a correlation between the body mass and the measured angle β in the transected and surgically repaired CrCL at a load of 30% BM.

## 4. Discussion

This study evaluated the use of a novel ultrasonically implanted absorbable polylactide bone anchor for fixation of fabellotibial suture in cats with CrCL rupture. The results partially confirmed our hypothesis, that using the fabellotibial suture technique with an ultrasonically implanted absorbable polylactide bone anchor can neutralize excessive craniocaudal movement and inward tibial rotation.

With our model, we showed that the use of the novel absorbable bone anchor neutralized the craniocaudal movement of the tibia based on conventional 2D measurement on radiographs but not at a load of 30% BM when calculating the corresponding angle. By contrast, the use of a nonabsorbable bone anchor for suture fixation failed to neutralize excessive cranial movement of the tibia according to 2D measurements, but this was not the case for 3D angle calculations. Internal tibial rotation was neutralized using all the techniques at all loads according to 2D and 3D measurements.

These conflicting results between measurements on radiographs representing craniocaudal movement of the tibia and angle α, which represents the same movement, lead to the suggestion that angle α may not properly reflect craniocaudal movement and may also yield false-positive results. This suspicion is supported by a recent study that suggested that a comparison between distance measurements and angle measurements using a CMM (Microscribe M; Revware Inc., Raleigh, NC, USA) is not possible, even if calculated distances are evaluated based on 3D measurements and not calculated angles [18].

To determine the possibility of comparing both methods, replication studies are recommended, keeping in mind that radiographs are the source used under clinical circumstances.

With regard to distance measurements, the differences between bone anchors may be due to different materials and implantation processes. The fact that the polylactide bone anchor liquefies during implantation, infiltrates bone cavities, and creates a rigid bond between bone and implant after immediate solidification might explain its superiority over the conventional screw-in anchor [20,21,28,31].

Another influencing factor might be the kind of suture attachment in different anchors. In the nonabsorbable anchor, the suture is not firmly attached and can become displaced after implantation, unlike the absorbable suture. This is because during ultrasonic implantation of an absorbable polylactide anchor, the material melts over the suture and creates an inextricable connection between anchor and suture, with the anchor itself rigidly bonding to the bone.

Regarding implantation, ultrasound-guided implantation of a polylactide anchor, in contrast to fabellotibial suture and nonabsorbable anchor placement, requires practice. Nevertheless, once the process of implantation is understood, the device is easy to use and does not require more time than that for conventional screw anchor placement. What needs to be considered is that the load needed for proper placement varies with the type of preloaded suture and must be higher when the suture material is braided and of a larger diameter. We experienced this during pretesting, when implantation was practiced using different suture materials. If excessive load is applied on the sonotrode during implantation, the anchor head breaks, as previously reported [19,20,21,31,36]. The same outcome can occur if the sonotrode is not placed straight on the anchor head during insertion.

Another phenomenon that needs to be considered when using absorbable polylactide anchors is the so-called backflow—i.e., when liquified material bulges out of the predrilled hole and creates a small bump on the bone surface. Backflow most likely occurs when the bone is not at the optimal temperature. This occurred in five of our tested hindlimbs and was resolved by immersing them in warm water for a few minutes prior to implantation to ensure body temperature inside the bone. Despite the backflow, all implants remained macroscopically stable and regained stability with regard to internal rotation and distance measurement. Although this phenomenon is unlikely to occur in live animals and may not lead to any soft-tissue irritation, it should be considered. Furthermore, the potential for irritation or even damage to the suture from protruding anchor material is not known and should be looked at in further studies. Regarding the increase in temperature during ultrasound-assisted implantation, no cellular reaction in the area around the pin has been found [19,20,21,31,36]. With regard to healing, different studies on the femur, tibia, jaw, and spine of sheep have shown high biocompatibility, with low scores or even the absence of inflammation-related cellular reactions [19,20,21,36]. To evaluate these findings in a clinical context, studies with long-term patient follow-up for tolerability of such implants are needed.

In our study, all the sutures were tied with the stifle joint held at 100°, as described in a study that reported that the tightening of lateral suture fixations results in the least loss of tension within that angle [34]. This study was performed on dogs with CrCL-deficient stifle joints, and the same angle was selected in previous studies on cats [16,18]. With regard to distance measurement in the G_FT_ group, craniocaudal instability neutralization was not possible within our setup where measurements were performed at a joint angle of 120 ± 5°. Considering this, further studies are necessary to prove whether suture tightening at a 100° angle is optimal in cats and with different surgical techniques. A force of 20 N was selected based on previous studies [16,18,33], but care needs to be taken because, to the best of our knowledge, no study has evaluated whether this force is most accurate for suture tightening in cats, and further evaluations are needed.

When interpreting our results, we were aware of the inherent limitations of an ex vivo biomechanical model. Such a model can never completely reflect the physiologic situation during hindlimb movement in live animals, especially as the functions of muscles and surrounding soft tissues in contributing to additional stability cannot be evaluated. The Achilles tendon, with its connection to the gastrocnemius, plays a major role in creating the cranial tibial thrust [37,38]. This leads to compression of the tibia against the femur and is neutralized by an intact CrCL, amongst other anatomical structures [38]. In the CrCL-deficient stifle joint, the Achilles tendon is responsible for excessive cranial translation of the tibia relative to the femur [37,38]. Even if we mimicked this anatomical structure in our experimental setting and a cranial and medial translation of the tibia was achieved within the CrCL-deficient stifle joints, the extent to which the ex vivo situation is able to reflect reality remains unclear. The contribution of creep due to repeated testing of the stifles and its possibility of altering the results should also be kept in mind.

Another important limitation was that testing was performed only at specific joint angles, making it impossible to explain the behavior of each kind of treatment during the various ranges of motion the joint experiences when a cat is walking. However, angles of stifle and hock joints were chosen as they occur in the early stance phase of a walking cat. This is the point where the stifle joint angle is the widest, which results in maximum instability for the CrCL [39,40]. A further limitation was that the maximum applied load was 30% BM. This load was supposed to mimic the load applied during normal walking of a cat but would be much lower than that required to accurately mimic a jumping cat. To avoid cable breakage and fixation loosening [16], it was not possible to apply higher loads in our setup, and this needs to be considered when interpreting the results. It can be assumed that any instability already visible at a load of 30% BM will be even more obvious with higher loads, as is the case, e.g., when a cat jumps.

For further clinical evaluation of sutures, it would be beneficial to perform cyclic testing of potential implant migration. At least, there was no possibility of evaluating postoperative clinical performance, including resolution of lameness, time of healing, and potential complications, including possible screw loosening or implant migration and, within that, a possible loss of stability.

The bias potentially created by our knowledge of which method was used when the radiographs were analyzed and coordinate measurements were performed is also a possible limiting factor.

## 5. Conclusions

The absorbable polylactide bone anchor tested in combination with a braided nonabsorbable multistrand polyethylene suture has the ability to neutralize excessive craniocaudal movement and internal tibial rotation following CrCL rupture in cats as demonstrated by distance measurements in radiographs for our ex vivo model and seems to be a suitable alternative to conventional suture anchors. Nevertheless, the conflicting results between distance measurements and angle calculations as well as the limitations of the experimental setup mean that further studies on this subject are necessary along with the consideration of possible limitations when interpreting the results.

## Figures and Tables

**Table 1:** Calculated differences in the distance measured for each load and each surgical treatment.

PBM ^5^	Distance Calculation	Lower	Upper	Mean (mm)	SD ^6^ (mm)	*p*-Value
10%	D_i_ ^1^ − D_FW_ ^2^	−0.1	0.4	0.1	0.4	0.29
	D_i_^1^ − D_FT_^3^	0.5	1.6	0.8	0.2	<0.01
	D_i_^1^ − D_WD_^4^	−0.0	0.3	0.1	0.3	0.10
20%	D_i_^1^ − D_FW_^2^	−0.2	0.3	0.1	0.4	0.52
	D_i_^1^ − D_FT_^3^	0.6	1.6	0.8	0.2	<0.01
	D_i_^1^ − D_WD_^4^	−0.1	0.4	0.2	0.4	0.18
30%	D_i_^1^ − D_FW_^2^	−0.2	0.3	0.0	0.4	0.75
	D_i_^1^ − D_FT_^3^	0.6	1.7	0.8	0.2	<0.01
	D_i_^1^ − D_WD_^4^	−0.0	0.4	0.2	0.3	0.07

^1^ D_i_ = distance measured in an intact cranial cruciate ligament. ^2^ D_FW_ = distance measured in stifle joint treated with #2 braided nonabsorbable multistrand polyethylene suture (FiberWire; Arthrex Vet Systems, Frechen, Germany) only. ^3^ D_FT_ = distance measured in stifle joint treated with nonabsorbable suture anchor (FASTak; Arthrex Vet Systems, Frechen, Germany) preloaded with #2 braided nonabsorbable multistrand polyethylene suture (FiberWire; Arthrex Vet Systems, Frechen, Germany). ^4^ D_WD_ = distance measured in stifle joint treated with polylactide absorbable bone anchor (Weldix; VetWelding AG, Stansstad, Switzerland) preloaded with #2 braided nonabsorbable multistrand polyethylene suture (FiberWire; Arthrex Vet Systems, Frechen, Germany). ^5^ PBM = percentage of body mass. ^6^ SD = standard deviation.

**Table 2:** Calculated differences in angle α, indicating craniocaudal movement, for each load and surgical treatment.

PBM ^5^	Angle Calculation	Lower	Upper	Mean (mm)	SD ^6^ (mm)	*p*-Value
10%	α_i_^1^ − α_FW_^2^	−7.7	2.3	−2.7	7.9	0.26
	α_i_^1^ − α_FT_^3^	−4.6	0.1	−2.2	3.6	0.06
	α_i_^1^ − α_WD_^4^	−3.5	1.1	−1.2	3.7	0.28
20%	α_i_^1^ − α_FW_^2^	−2.2	3.2	0.5	4.3	0.67
	α_i_^1^ − α_FT_^3^	−4.3	0.7	−1.8	3.9	0.14
	α_i_^1^ − α_WD_^4^	−4.4	1.4	−1.5	4.5	0.27
30%	α_i_^1^ − α_FW_^2^	−5.3	−0.1	−2.7	4.2	0.05
	α_i_^1^ − α_FT_^3^	−4.8	0.1	−2.4	3.8	0.06
	α_i_^1^ − α_WD_^4^	−6.2	−0.3	−3.2	4.7	0.04

^1^ α_i_ = distance measured in intact cranial cruciate ligament. ^2^ α_FW_ = distance measured in stifle joint treated with #2 braided nonabsorbable multistrand polyethylene suture (FiberWire; Arthrex Vet Systems, Frechen, Germany) only. ^3^ α_FT_ = distance measured in stifle joint treated with nonabsorbable suture anchor (FASTak; Arthrex Vet Systems, Frechen, Germany) preloaded with #2 braided nonabsorbable multistrand polyethylene suture (FiberWire; Arthrex Vet Systems, Frechen, Germany). ^4^ α_WD_ = distance measured in stifle joint treated with polylactide absorbable bone anchor (Weldix; VetWelding AG, Stansstad, Switzerland) preloaded with #2 braided nonabsorbable multistrand polyethylene suture (FiberWire; Arthrex Vet Systems, Frechen, Germany). ^5^ PBM = percentage of body mass. ^6^ SD = standard deviation.

**Table 3:** Calculated differences of angle β, indicating internal rotation of the tibia, for each load and surgical treatment.

PBM ^5^	Distance Calculation	Lower	Upper	Mean (mm)	SD ^6^ (mm)	*p*-Value
10%	β_i_^1^ − β_FW_^2^	−6.6	13.2	3.3	15.5	0.48
	β_i_^1^ − β_FT_^3^	−4.6	0.1	−2.2	3.6	0.06
	β_i_^1^ − β_WD_^4^	−11.1	3.0	−4.0	11.1	0.24
20%	β_i_^1^ − β_FW_^2^	−10.0	7.0	−1.5	13.4	0.71
	β_i_^1^ − β_FT_^3^	−8.1	2.6	−2.8	8.4	0.28
	β_i_^1^ − β_WD_^4^	−4.6	0.8	−1.9	4.2	0.15
30%	β_i_^1^ − β_FW_^2^	−8.2	11.7	1.8	15.7	0.71
	β_i_^1^ − β_FT_^3^	−6.0	6.1	0.1	9.5	0.98
	β_i_^1^ − β_WD_^4^	−5.5	7.0	0.7	9.8	0.80

^1^ βi = distance measured in intact cranial cruciate ligament. ^2^ β_FW_ = distance measured in stifle joint treated with #2 braided nonabsorbable multistrand polyethylene suture (FiberWire; Arthrex Vet Systems, Frechen, Germany) only. ^3^ β_FT_ = distance measured in stifle joint treated with nonabsorbable suture anchor (FASTak; Arthrex Vet Systems, Frechen, Germany) preloaded with #2 braided nonabsorbable multistrand polyethylene suture (FiberWire; Arthrex Vet Systems, Frechen, Germany). ^4^ β_WD_ = distance measured in stifle joint treated with polylactide absorbable bone anchor (Weldix; VetWelding AG, Stansstad, Switzerland) preloaded with #2 braided nonabsorbable multistrand polyethylene suture (FiberWire; Arthrex Vet Systems, Frechen, Germany).^5^ PBM = percentage of body mass. ^6^ SD = standard deviation.

## Data Availability

The data presented in this study are available on request from the corresponding author.

## Supplementary Materials

The following are available online at https://www.mdpi.com/article/10.3390/ani11061695/s1, Tables S1–S27: All calculated distances and angles, including percentages of body mass and patellar tendon load, for each cat and each loading.
