# Supplementary material for: Comparison of Extracapsular Stabilization Techniques Using an Ultrasonically Implanted Absorbable Bone Anchor (Weldix) after Cranial Cruciate Ligament Rupture in Cats—An In Vitro Study
Source: Animals (Basel). 2021 Jun 7;11(6):1695. doi: 10.3390/ani11061695 (PMC8227609; doi:10.3390/ani11061695)
Supplement: Supplementary file 1 [file animals-11-01695-s001.zip › animals-1223991-supplementary.pdf]

Table S1. Body mass (BM), percentage BM , patellar tendon load, mesured distances on radiographs and calculated angles for intact and transected CrCL and after surgically treatment of cat 1.

CAT 1							
**BM (g)**	**10% BM (g)**	**20% BM (g)**	**30% BM (g)**	**PTL 10% BM**	**PTL 20% BM**	**PTL 30% BM**	
2030	203	406	609	13,8	14,5	15,3	
**RIGHT - FT**				**LEFT**			
**measured distances radiographs (cm)**				REJECTED - FRACTURE OF TIBIA			
PBM	INTACT	TRANSECTED	TREATED				
10%	8	11,3	8,6				
20%	8,1	11,6	8,9				
30%	8,3	12,1	9,3				
**calculated angle alpha (degrees)**							
PBM	INTACT	TRANSECTED	TREATED				
10%	68,3	63,7	71,3				
20%	67,4	63,8	70,7				
30%	66,7	59,8	70,2				
**calculated angle beta (degrees)**							
PBM	INTACT	TRANSECTED	TREATED				
10%	64,9	79,6	54,7				
20%	71,2	85,4	58,2				
30%	72,6	94,2	53,3				

Table S2. Body mass (BM), percentage BM , patellar tendon load, mesured distances on radiographs and calculated angles for intact and transected CrCL and after surgically treatment of cat 2.

CAT 2							
**BM**	**10% BM**	**20% BM**	**30% BM**	**PTL 10% BM**	**PTL 20% BM**	**PTL 30% BM**	
2840	284	568	852	21,2	20,2	22,3	
**RIGHT - WD**				**LEFT - FW**			
**measured distances radiographs (cm)**				**measured distances radiographs (cm)**			
PBM	INTACT	TRANSECTED	TREATED	PBM	INTACT	TRANSECTED	TREATED
10%	8,6	13,8	8,5	10%	8,7	13,4	7,6
20%	8,8	13,4	8,4	20%	8,8	13,1	7,8
30%	8,9	14,3	8,5	30%	9,1	13,7	7,8
**calculated angle alpha (degrees)**				**calculated angle alpha (degrees)**			
PBM	INTACT	TRANSECTED	TREATED	PBM	INTACT	TRANSECTED	TREATED
10%	68,9	59,1	73,9	10%	74,2	64,7	79,8
20%	68,4	61,7	74,1	20%	72,7	66,1	75,7
30%	71,8	55,7	75,4	30%	73,3	64,2	79,3
**calculated angle beta (degrees)**				**calculated angle beta (degrees)**			
PBM	INTACT	TRANSECTED	TREATED	PBM	INTACT	TRANSECTED	TREATED
10%	50,2	88,4	43,6	10%	42,6	85,8	43,3
20%	48,6	84,5	49,2	20%	47,2	81,4	44,7
30%	45,7	80,3	50,9	30%	47,6	96,3	40,6

Table S3. Body mass (BM), percentage BM , patellar tendon load, mesured distances on radiographs and calculated angles for intact and transected CrCL and after surgically treatment of cat 3.

CAT 3							
**BM**	**10% BM**	**20% BM**	**30% BM**	**PTL 10% BM**	**PTL 20% BM**	**PTL 30% BM**	
3300	330	660	990	15,8	17,3	18,0	
**RIGHT - FT**				**LEFT - FT**			
**measured distances radiographs (cm)**				**measured distances radiographs (cm)**			
PBM	INTACT	TRANSECTED	TREATED	PBM	INTACT	TRANSECTED	TREATED
10%	8,7	11,1	9,7	10%	8,5	12,3	10
20%	8,8	11,5	10	20%	8,8	12,3	10,1
30%	9	11,8	10	30%	8,9	14	10,1
**calculated angle alpha (degrees)**				**calculated angle alpha (degrees)**			
PBM	INTACT	TRANSECTED	TREATED	PBM	INTACT	TRANSECTED	TREATED
10%	69,4	66,5	69,3	10%	78,1	67,6	73,2
20%	70,4	67,0	71,1	20%	74,6	62,5	72,3
30%	70,0	64,6	69,6	30%	75,3	63,8	73,0
**calculated angle beta (degrees)**				**calculated angle beta (degrees)**			
PBM	INTACT	TRANSECTED	TREATED	PBM	INTACT	TRANSECTED	TREATED
10%	55,8	71,9	60,4	10%	46,5	81,5	39,8
20%	60,5	69,7	58,0	20%	53,2	81,6	41,7
30%	54,6	79,3	61,5	30%	49,2	91,1	38,8

Table S4. Body mass (BM), percentage BM , patellar tendon load, mesured distances on radiographs and calculated angles for intact and transected CrCL and after surgically treatment of cat 4.

CAT 4							
**BM**	**10% BM**	**20% BM**	**30% BM**	**PTL 10% BM**	**PTL 20% BM**	**PTL 30% BM**	
4130	413	826	1239	15,0	15,2	16,3	
**RIGHT - FT**				**LEFT - FW**			
**measured distances radiographs (cm)**				**measured distances radiographs (cm)**			
PBM	INTACT	TRANSECTED	TREATED	PBM	INTACT	TRANSECTED	TREATED
10%	9,5	15,7	12,3	10%	8,9	15,4	9,1
20%	9,5	15,8	12,5	20%	9,1	15,5	9,5
30%	9,6	16,6	12,6	30%	9,2	16	9,5
**calculated angle alpha (degrees)**				**calculated angle alpha (degrees)**			
PBM	INTACT	TRANSECTED	TREATED	PBM	INTACT	TRANSECTED	TREATED
10%	68,5	56,3	63,1	10%	70,3	60,5	46,4
20%	67,4	58,3	64,2	20%	68,2	57,5	71,6
30%	66,0	53,3	62,5	30%	70,0	57,0	68,8
**calculated angle beta (degrees)**				**calculated angle beta (degrees)**			
PBM	INTACT	TRANSECTED	TREATED	PBM	INTACT	TRANSECTED	TREATED
10%	62,1	117,1	64,5	10%	43,2	91,0	39,2
20%	65,0	110,4	68,9	20%	44,7	48,3	45,9
30%	63,6	120,3	65,9	30%	44,2	94,7	40,4

Table S5. Body mass (BM), percentage BM , patellar tendon load, mesured distances on radiographs and calculated angles for intact and transected CrCL and after surgically treatment of cat 5.

CAT 5							
**BM**	**10% BM**	**20% BM**	**30% BM**	**PTL 10% BM**	**PTL 20% BM**	**PTL 30% BM**	
3740	374	748	1122	14,3	15,8	16,5	
**RIGHT - FT**				**LEFT**			
**measured distances radiographs (cm)**							
PBM	INTACT	TRANSECTED	TREATED				
10%	8,7	12,3	10,4				
20%	8,8	12	10,7				
30%	8,8	13,3	10,8				
**calculated angle alpha (degrees)**							
PBM	INTACT	TRANSECTED	TREATED				
10%	69,9	59,3	65,3	**REJECTED - FRACTURE OF FEMUR DURING PREPERATION**			
20%	69,9	60,6	63,8				
30%	69,6	58,5	64,0				
**calculated angle beta (degrees)**							
PBM	INTACT	TRANSECTED	TREATED				
10%	58,6	90,2	66,5				
20%	50,3	91,9	62,1				
30%	50,3	97,2	64,5				

Table S6. Body mass (BM), percentage BM , patellar tendon load, mesured distances on radiographs and calculated angles for intact and transected CrCL and after surgically treatment of cat 6.

CAT 6							
**BM**	**10% BM**	**20% BM**	**30% BM**	**PTL 10% BM**	**PTL 20% BM**	**PTL 30% BM**	
2780	278	556	834	10,5	11,5	11,3	
**RIGHT - WD**				**LEFT**			
**measured distances radiographs (cm)**							
PBM	INTACT	TRANSECTED	TREATED				
10%	9,6	14,9	9,6				
20%	9,9	15,3	10,7				
30%	10,1	15,6	10,7				
**calculated angle alpha (degrees)**							
PBM	INTACT	TRANSECTED	TREATED				
10%	73,1	62,7	72,6	**REJECTED - FEMORAL FRACTURE DURING PREPERATION**			
20%	73,8	62,6	70,3				
30%	73,7	58,9	65,9				
**calculated angle beta (degrees)**							
PBM	INTACT	TRANSECTED	TREATED				
10%	56,5	92,1	42,4				
20%	57,1	97,6	49,8				
30%	56,0	101,4	48,5				

Table S7. Body mass (BM), percentage BM , patellar tendon load, mesured distances on radiographs and calculated angles for intact and transected CrCL and after surgically treatment of cat 7.

CAT 7							
**BM**	**10% BM**	**20% BM**	**30% BM**	**PTL 10% BM**	**PTL 20% BM**	**PTL 30% BM**	
5280	528	1056	1584	19,7	19,2	20,7	
**RIGHT - WD**				**LEFT - WD**			
**measured distances radiographs (cm)**				**measured distances radiographs (cm)**			
PBM	INTACT	TRANSECTED	TREATED	PBM	INTACT	TRANSECTED	TREATED
10%	8,5	12,6	8,6	10%	8,1	12	8
20%	8,7	12,5	8,8	20%	8,5	12,1	8,5
30%	8,9	13,2	9,4	30%	8,5	12,8	8,5
**calculated angle alpha (degrees)**				**calculated angle alpha (degrees)**			
PBM	INTACT	TRANSECTED	TREATED	PBM	INTACT	TRANSECTED	TREATED
10%	76,4	65,6	72,8	10%	76,4	65,8	75,9
20%	72,9	63,7	75,4	20%	71,2	65,5	73,8
30%	75,7	62,2	71,8	30%	74,4	63,1	72,2
**calculated angle beta (degrees)**				**calculated angle beta (degrees)**			
PBM	INTACT	TRANSECTED	TREATED	PBM	INTACT	TRANSECTED	TREATED
10%	51,1	80,2	33,5	10%	42,9	81,4	51,8
20%	48,5	97,6	54,9	20%	47,7	82,1	45,3
30%	49,2	124,4	41,2	30%	45,0	90,3	52,0

Table S8. Body mass (BM), percentage BM , patellar tendon load, mesured distances on radiographs and calculated angles for intact and transected CrCL and after surgically treatment of cat 8.

CAT 8							
**BM**	**10% BM**	**20% BM**	**30% BM**	**PTL 10% BM**	**PTL 20% BM**	**PTL 30% BM**	
3650	365	730	1095	20,7	25,8	30,2	
**RIGHT - WD**				**LEFT - WD**			
**measured distances radiographs (cm)**				**measured distances radiographs (cm)**			
PBM	INTACT	TRANSECTED	TREATED	PBM	INTACT	TRANSECTED	TREATED
10%	9,9	14,1	10	10%	9,7	13,1	9,7
20%	10,1	14,1	10,2	20%	9,8	13,4	9,8
30%	10,5	15,3	10,7	30%	9,8	13,8	9,9
**calculated angle alpha (degrees)**				**calculated angle alpha (degrees)**			
PBM	INTACT	TRANSECTED	TREATED	PBM	INTACT	TRANSECTED	TREATED
10%	73,1	63,5	71,7	10%	70,2	62,1	72,4
20%	77,9	66,5	70,4	20%	68,4	58,9	67,0
30%	75,0	62,0	71,2	30%	69,5	59,4	68,2
**calculated angle beta (degrees)**				**calculated angle beta (degrees)**			
PBM	INTACT	TRANSECTED	TREATED	PBM	INTACT	TRANSECTED	TREATED
10%	49,4	58,5	47,4	10%	42,0	72,4	43,0
20%	49,7	67,2	47,9	20%	38,8	68,7	36,6
30%	51,9	82,2	50,4	30%	42,9	82,6	39,0

Table S9. Resons for rejection of both hindlimbs of cat 9.

CAT 9	
**RIGHT**	**LEFT**
**REJECTED - Ankylosis in tarsal joint**	**REJECTED - excessive rotation on radiograph**

Table S10. Body mass (BM), percentage BM , patellar tendon load, mesured distances on radiographs and calculated angles for intact and transected CrCL and after surgically treatment of cat 10.

CAT 10							
**BM**	**10% BM**	**20% BM**	**30% BM**	**PTL 10% BM**	**PTL 20% BM**	**PTL 30% BM**	
3270	327	654	981	12,2	14,3	15,3	
**RECHTS - FT**				**LINKS - WD**			
**measured distances radiographs (cm)**				**measured distances radiographs (cm)**			
PBM	INTACT	TRANSECTED	TREATED	PBM	INTACT	TRANSECTED	TREATED
10%	8,2	12,9	8,8	10%	7,8	11,4	8
20%	8,3	13,1	8,9	20%	8,2	12,2	8,2
30%	8,3	13,3	9,3	30%	8,3	12,2	8,5
**calculated angle alpha (degrees)**				**calculated angle alpha (degrees)**			
PBM	INTACT	TRANSECTED	TREATED	PBM	INTACT	TRANSECTED	TREATED
10%	76,0	68,1	74,2	10%	74,5	66,0	77,2
20%	77,4	67,4	71,7	20%	74,4	66,1	76,6
30%	76,1	66,9	72,3	30%	73,6	63,5	74,7
**calculated angle beta (degrees)**				**calculated angle beta (degrees)**			
PBM	INTACT	TRANSECTED	TREATED	PBM	INTACT	TRANSECTED	TREATED
10%	48,7	91,9	48,8	10%	42,1	80,4	44,4
20%	51,8	91,5	58,2	20%	45,2	67,5	48,1
30%	49,0	99,9	52,0	30%	46,4	88,6	47,9

Table S11. Body mass (BM), percentage BM , patellar tendon load, mesured distances on radiographs and calculated angles for intact and transected CrCL and after surgically treatment of cat 11.

CAT 11							
**BM**	**10% BM**	**20% BM**	**30% BM**	**PTL 10% BM**	**PTL 20% BM**	**PTL 30% BM**	
4900	490	980	1470	20,5	23,2	25,3	
**RECHTS - WD**				**LINKS**			
**measured distances radiographs (cm)**							
PBM	INTACT	TRANSECTED	TREATED				
10%	9,8	13,1	10,7				
20%	9,8	13,4	10,7				
30%	10	14,3	10,9				
**calculated angle alpha (degrees)**							
PBM	INTACT	TRANSECTED	TREATED				
10%	75,7	64,3	67,7	**REJECTED - DOUBLE CONDYLAR SIGN ON RX**			
20%	75,0	67,5	67,8				
30%	79,7	63,8	64,7				
**calculated angle beta (degrees)**							
PBM	INTACT	TRANSECTED	TREATED				
10%	47,1	87,7	62,3				
20%	67,0	87,6	66,8				
30%	44,3	99,2	71,5				

Table S12. Body mass (BM), percentage BM , patellar tendon load, mesured distances on radiographs and calculated angles for intact and transected CrCL and after surgically treatment of cat 12.

CAT 12							
**BM**	**10% BM**	**20% BM**	**30% BM**	**PTL 10% BM**	**PTL 20% BM**	**PTL 30% BM**	
3030	303	606	909	18,3	12,5	21,0	
**RECHTS - FT**				**LINKS - FT**			
**measured distances radiographs (cm)**				**measured distances radiographs (cm)**			
PBM	INTACT	TRANSECTED	TREATED	PBM	INTACT	TRANSECTED	TREATED
10%	8,8	12,4	9,2	10%	9	12,4	9,1
20%	9,2	12,5	9,1	20%	9	12,4	9,6
30%	9,3	13,5	9,1	30%	9,2	13,3	9,6
**calculated angle alpha (degrees)**				**calculated angle alpha (degrees)**			
PBM	INTACT	TRANSECTED	TREATED	PBM	INTACT	TRANSECTED	TREATED
10%	76,7	64,9	76,2	10%	72,5	62,7	76,4
20%	76,7	62,2	78,9	20%	75,4	60,6	80,1
30%	74,6	64,1	75,6	30%	72,9	58,5	75,4
**calculated angle beta (degrees)**				**calculated angle beta (degrees)**			
PBM	INTACT	TRANSECTED	TREATED	PBM	INTACT	TRANSECTED	TREATED
10%	62,7	115,6	58,2	10%	53,2	104,8	47,7
20%	66,2	113,8	57,4	20%	53,0	101,1	44,3
30%	63,8	120,3	55,8	30%	49,7	108,0	46,8

Table S13. Body mass (BM), percentage BM , patellar tendon load, mesured distances on radiographs and calculated angles for intact and transected CrCL and after surgically treatment of cat 13.

CAT 13							
**BM**	**10% BM**	**20% BM**	**30% BM**	**PTL 10% BM**	**PTL 20% BM**	**PTL 30% BM**	
5000	500	1000	1500	24,8	24,7	30,0	
**RECHTS**				**LINKS - FW**			
**REJECTED - ROTATION OF FEMUR IN COPPER TUBE**				**measured distances radiographs (cm)**			
				PBM	INTACT	TRANSECTED	TREATED
				10%	9,9	14,3	10,5
				20%	10,1	15	10,7
				30%	10,3	15,9	10,7
				**calculated angle alpha (degrees)**			
				PBM	INTACT	TRANSECTED	TREATED
				10%	70,6	70,5	73,8
				20%	68,9	67,5	74,3
				30%	74,3	67,2	71,7
				**calculated angle beta (degrees)**			
				PBM	INTACT	TRANSECTED	TREATED
				10%	51,1	78,0	59,6
				20%	51,8	58,3	55,2
				30%	50,2	89,1	58,3

Table S14. Body mass (BM), percentage BM , patellar tendon load, mesured distances on radiographs and calculated angles for intact and transected CrCL and after surgically treatment of cat 14.

CAT 14							
**BM**	**10% BM**	**20% BM**	**30% BM**	**PTL 10% BM**	**PTL 20% BM**	**PTL 30% BM**	
2700	270	540	810	20,3	20,0	23,5	
**RECHTS - FW**				**LINKS - FW**			
**measured distances radiographs (cm)**				**measured distances radiographs (cm)**			
PBM	INTACT	TRANSECTED	TREATED	PBM	INTACT	TRANSECTED	TREATED
10%	8,6	10,1	8,7	10%	10	14,3	10,1
20%	9,5	10,7	9,5	20%	10,1	15,2	10,1
30%	9,5	11,3	9,4	30%	10,5	15,3	10,6
**calculated angle alpha (degrees)**				**calculated angle alpha (degrees)**			
PBM	INTACT	TRANSECTED	TREATED	PBM	INTACT	TRANSECTED	TREATED
10%	81,2	80,7	76,8	10%	74,1	67,4	72,7
20%	78,8	75,3	75,8	20%	74,8	64,2	68,6
30%	79,4	68,8	73,9	30%	73,2	61,4	67,0
**calculated angle beta (degrees)**				**calculated angle beta (degrees)**			
PBM	INTACT	TRANSECTED	TREATED	PBM	INTACT	TRANSECTED	TREATED
10%	29,1	34,4	33,8	10%	48,9	74,2	49,8
20%	38,7	36,8	39,8	20%	24,2	82,7	53,3
30%	29,6	59,8	45,8	30%	60,7	104,6	54,9

Table S15. Body mass (BM), percentage BM , patellar tendon load, mesured distances on radiographs and calculated angles for intact and transected CrCL and after surgically treatment of cat 15.

CAT 15							
**BM**	**10% BM**	**20% BM**	**30% BM**	**PTL 10% BM**	**PTL 20% BM**	**PTL 30% BM**	
5350	535	1070	1605	15,7	19,3	21,3	
**RECHTS - WD**				**LINKS**			
**measured distances radiographs (cm)**				**REJECTED - TIBIAL FRCTURE**			
PBM	INTACT	TRANSECTED	TREATED				
10%	9,2	14,4	9,4				
20%	9,8	14,6	9,8				
30%	9,8	15,7	9,8				
**calculated angle alpha (degrees)**							
PBM	INTACT	TRANSECTED	TREATED				
10%	75,4	65,8	70,2				
20%	76,6	64,5	71,4				
30%	75,3	61,6	70,5				
**calculated angle beta (degrees)**							
PBM	INTACT	TRANSECTED	TREATED				
10%	54,2	81,4	45,4				
20%	54,7	115,3	50,9				
30%	51,1	117,1	49,1				

Table S16. Body mass (BM), percentage BM , patellar tendon load, mesured distances on radiographs and calculated angles for intact and transected CrCL and after surgically treatment of cat 16.

CAT 16							
**BM**	**10% BM**	**20% BM**	**30% BM**	**PTL 10% BM**	**PTL 20% BM**	**PTL 30% BM**	
3920	392	784	1176	15,3	15,7	16,0	
**RECHTS - FT**				**LINKS - WD**			
**measured distances radiographs (cm)**				**measured distances radiographs (cm)**			
PBM	INTACT	TRANSECTED	TREATED	PBM	INTACT	TRANSECTED	TREATED
10%	8,5	12	9,7	10%	8,8	11,9	9,1
20%	8,5	12,1	10,2	20%	8,8	12,2	9,1
30%	8,9	12,9	10,2	30%	9	12,9	9,3
**calculated angle alpha (degrees)**				**calculated angle alpha (degrees)**			
PBM	INTACT	TRANSECTED	TREATED	PBM	INTACT	TRANSECTED	TREATED
10%	71,3	59,3	67,7	10%	72,2	61,3	73,4
20%	68,6	60,4	68,9	20%	71,5	63,9	74,5
30%	70,4	58,7	67,6	30%	73,3	63,3	72,2
**calculated angle beta (degrees)**				**calculated angle beta (degrees)**			
PBM	INTACT	TRANSECTED	TREATED	PBM	INTACT	TRANSECTED	TREATED
10%	64,3	106,2	63,4	10%	48,0	85,2	53,1
20%	76,2	101,4	62,5	20%	49,9	82,8	47,1
30%	65,9	110,4	63,0	30%	50,4	93,4	52,9

Table S17. Body mass (BM), percentage BM , patellar tendon load, mesured distances on radiographs and calculated angles for intact and transected CrCL and after surgically treatment of cat 17.

CAT 17							
**BM**	**10% BM**	**20% BM**	**30% BM**	**PTL 10% BM**	**PTL 20% BM**	**PTL 30% BM**	
5330	533	1066	1599	25,7	26,5	26,3	
**RECHTS - FW**				**LINKS - WD**			
**measured distances radiographs (cm)**				**measured distances radiographs (cm)**			
PBM	INTACT	TRANSECTED	TREATED	PBM	INTACT	TRANSECTED	TREATED
10%	10,3	15	10,6	10%	10,1	14,2	10
20%	10,3	15	10,3	20%	10,1	14	10
30%	10,6	16,2	10,7	30%	10,3	14,8	10,3
**calculated angle alpha (degrees)**				**calculated angle alpha (degrees)**			
PBM	INTACT	TRANSECTED	TREATED	PBM	INTACT	TRANSECTED	TREATED
10%	70,3	63,3	71,5	10%	71,7	56,5	69,1
20%	68,6	60,5	74,2	20%	70,9	61,1	67,0
30%	70,5	55,6	71,4	30%	70,6	53,2	68,9
**calculated angle beta (degrees)**				**calculated angle beta (degrees)**			
PBM	INTACT	TRANSECTED	TREATED	PBM	INTACT	TRANSECTED	TREATED
10%	56,4	80,5	51,4	10%	46,1	83,0	36,3
20%	56,0	107,3	52,6	20%	47,7	74,6	38,2
30%	63,9	120,6	52,2	30%	46,2	101,6	36,5

Table S18. Resons for rejection of both hindlimbs of cat 18.

CAT 18	
**RECHTS**	**LEFT**
**REJECTED - CAUDAL DRAWER TEST POSITIVE**	**REJECTED - WIRE IN TIBIAL TUBEROSITY**

Table S19. Body mass (BM), percentage BM , patellar tendon load, mesured distances on radiographs and calculated angles for intact and transected CrCL and after surgically treatment of cat 19.

CAT 19							
**BM**	**10% BM**	**20% BM**	**30% BM**	**PTL 10% BM**	**PTL 20% BM**	**PTL 30% BM**	
2990	299	598	897	21,7	26,0	31,3	
**RECHTS - FW**				**LINKS - FW**			
**measured distances radiographs (cm)**				**measured distances radiographs (cm)**			
PBM	INTACT	TRANSECTED	TREATED	PBM	INTACT	TRANSECTED	TREATED
10%	9,1	12,9	9,4	10%	9,1	12,5	9,2
20%	9,4	13,3	9,5	20%	9,3	12,7	9,6
30%	9,6	13,8	9,8	30%	9,5	13,2	9,7
**calculated angle alpha (degrees)**				**calculated angle alpha (degrees)**			
PBM	INTACT	TRANSECTED	TREATED	PBM	INTACT	TRANSECTED	TREATED
10%	74,7	70,9	77,8	10%	74,8	67,0	77,5
20%	78,0	68,3	75,6	20%	72,0	63,5	79,4
30%	81,1	64,4	73,7	30%	72,1	39,7	75,1
**calculated angle beta (degrees)**				**calculated angle beta (degrees)**			
PBM	INTACT	TRANSECTED	TREATED	PBM	INTACT	TRANSECTED	TREATED
10%	50,0	23,1	32,2	10%	49,8	89,9	53,2
20%	26,6	49,6	28,9	20%	51,5	91,8	46,0
30%	42,2	51,7	46,8	30%	51,0	107,9	45,8

Table S20. Body mass (BM), percentage BM , patellar tendon load, mesured distances on radiographs and calculated angles for intact and transected CrCL and after surgically treatment of cat 20.

CAT 20							
**BM**	**10% BM**	**20% BM**	**30% BM**	**PTL 10% BM**	**PTL 20% BM**	**PTL 30% BM**	
6160	616	1232	1848	16,0	19,2	19,7	
**RIGHT**				**LINKS - FT**			
				**measured distances radiographs (cm)**			
				PBM	INTACT	TRANSECTED	TREATED
				10%	10,1	14,2	10,5
				20%	10,3	15,3	10,9
				30%	10,3	15,4	10,9
				**calculated angle alpha (degrees)**			
				PBM	INTACT	TRANSECTED	TREATED
				10%	73,5	58,1	68,6
				20%	73,4	61,4	68,1
				30%	73,9	58,2	69,2
				**calculated angle beta (degrees)**			
				PBM	INTACT	TRANSECTED	TREATED
				10%	41,1	88,9	43,5
				20%	39,3	83,4	40,4
				30%	41,6	108,6	48,1

Table S21. Body mass (BM), percentage BM , patellar tendon load, mesured distances on radiographs and calculated angles for intact and transected CrCL and after surgically treatment of cat 21.

CAT 21							
**BM**	**10% BM**	**20% BM**	**30% BM**	**PTL 10% BM**	**PTL 20% BM**	**PTL 30% BM**	
2650	265	530	795	13,2	11,0	15,7	
**RECHTS - FW**				**LINKS - WD**			
**measured distances radiographs (cm)**				**measured distances radiographs (cm)**			
PBM	INTACT	TRANSECTED	TREATED	PBM	INTACT	TRANSECTED	TREATED
10%	8,4	12,4	8,6	10%	8,2	12,2	8,4
20%	8,6	12,8	8,8	20%	8,4	12,2	8,5
30%	8,7	13	8,9	30%	8,5	12,7	8,5
**calculated angle alpha (degrees)**				**calculated angle alpha (degrees)**			
PBM	INTACT	TRANSECTED	TREATED	PBM	INTACT	TRANSECTED	TREATED
10%	68,8	60,3	65,4	10%	68,8	56,3	65,1
20%	66,7	60,0	64,6	20%	67,8	55,6	62,4
30%	69,4	57,8	64,5	30%	65,1	51,2	63,2
**calculated angle beta (degrees)**				**calculated angle beta (degrees)**			
PBM	INTACT	TRANSECTED	TREATED	PBM	INTACT	TRANSECTED	TREATED
10%	50,0	88,2	49,0	10%	49,9	104,3	28,0
20%	58,1	116,3	45,4	20%	42,6	93,0	39,9
30%	54,9	94,7	47,4	30%	39,6	90,4	37,5

Table S22. Body mass (BM), percentage BM , patellar tendon load, mesured distances on radiographs and calculated angles for intact and transected CrCL and after surgically treatment of cat 22.

CAT 22							
**BM**	**10% BM**	**20% BM**	**30% BM**	**PTL 10% BM**	**PTL 20% BM**	**PTL 30% BM**	
2210	221	442	663	4,7	10,0	9,7	
**RECHTS - FW**				**LINKS**			
**measured distances radiographs (cm)**							
PBM	INTACT	TRANSECTED	TREATED				
10%	8,5	13,4	8,8				
20%	9	14,1	9				
30%	9,1	14,2	9,1				
**calculated angle alpha (degrees)**							
PBM	INTACT	TRANSECTED	TREATED				
10%	81,3	70,1	71,7				
20%	79,1	69,5	77,1				
30%	83,2	68,3	76,6				
**calculated angle beta (degrees)**							
PBM	INTACT	TRANSECTED	TREATED				
10%	20,8	64,8	68,2				
20%	36,9	48,9	46,1				
30%	28,2	57,6	43,2				

Table S23. Body mass (BM), percentage BM , patellar tendon load, mesured distances on radiographs and calculated angles for intact and transected CrCL and after surgically treatment of cat 23.

CAT 23							
**BM**	**10% BM**	**20% BM**	**30% BM**	**PTL 10% BM**	**PTL 20% BM**	**PTL 30% BM**	
3000	300	600	900	13,0	15,0	14,7	
**RIGHT**				**LINKS - FT**			
				**measured distances radiographs (cm)**			
				PBM	INTACT	TRANSECTED	TREATED
				10%	9,9	13,3	10,3
				20%	9,9	14,2	10,3
				30%	10	14,4	10,5
				**calculated angle alpha (degrees)**			
				PBM	INTACT	TRANSECTED	TREATED
				10%	72,7	64,8	73,0
				20%	75,1	63,4	70,9
				30%	74,5	60,8	72,7
				**calculated angle beta (degrees)**			
				PBM	INTACT	TRANSECTED	TREATED
				10%	41,7	66,5	36,6
				20%	40,3	68,3	38,6
				30%	39,7	78,6	39,7

Table S24. Body mass (BM), percentage BM , patellar tendon load, mesured distances on radiographs and calculated angles for intact and transected CrCL and after surgically treatment of cat 24.

CAT 24							
**BM**	**10% BM**	**20% BM**	**30% BM**	**PTL 10% BM**	**PTL 20% BM**	**PTL 30% BM**	
2890	289	578	867	14,2	13,8	16,7	
**RECHTS - FT**				**LINKS**			
**measured distances radiographs (cm)**							
PBM	INTACT	TRANSECTED	TREATED				
10%	8,1	12,4	10,1				
20%	8,5	12,5	9,8				
30%	8,6	12,8	10,3				
**calculated angle alpha (degrees)**							
PBM	INTACT	TRANSECTED	TREATED				
10%	75,9	63,4	67,7				
20%	76,2	63,0	70,2				
30%	76,5	62,8	65,9				
**calculated angle beta (degrees)**							
PBM	INTACT	TRANSECTED	TREATED				
10%	58,8	96,1	72,8				
20%	60,8	106,0	64,4				
30%	60,2	105,8	71,5				

Table S25. Body mass (BM), percentage BM , patellar tendon load, mesured distances on radiographs and calculated angles for intact and transected CrCL and after surgically treatment of cat 25.

CAT 25							
**BM**	**10% BM**	**20% BM**	**30% BM**	**PTL 10% BM**	**PTL 20% BM**	**PTL 30% BM**	
4280	428	856	1284	20,5	18,5	20,5	
**RECHTS**				**LINKS - FW**			
				**measured distances radiographs (cm)**			
				PBM	INTACT	TRANSECTED	TREATED
				10%	8,4	12,4	8,6
				20%	8,6	12,6	8,7
				30%	8,6	12,7	8,7
				**calculated angle alpha (degrees)**			
				PBM	INTACT	DISS.	TREATED
				10%	72,1	61,2	68,4
				20%	70,6	60,8	68,0
				30%	72,2	59,2	67,4
				**calculated angle beta (degrees)**			
				PBM	INTACT	TRANSECTED	TREATED
				10%	43,1	74,7	49,7
				20%	46,7	97,6	43,6
				30%	44,4	96,7	44,1

Table S26. Body mass (BM), percentage BM , patellar tendon load, mesured distances on radiographs and calculated angles for intact and transected CrCL and after surgically treatment of cat 26.

CAT 26							
**BM**	**10% BM**	**20% BM**	**30% BM**	**PTL 10% BM**	**PTL 20% BM**	**PTL 30% BM**	
3710	371	742	1113	10,0	15,5	17,2	
**RECHTS - FW**				**LINKS**			
**measured distances radiographs (cm)**							
PBM	INTACT	TRANSECTED	TREATED				
10%	8,6	12,5	8,9				
20%	8,8	13	9				
30%	9	13,2	9,3				
**calculated angle alpha (degrees)**							
PBM	INTACT	TRANSECTED	TREATED				
10%	70,7	62,5	69,0				
20%	69,7	61,9	69,6				
30%	70,5	62,0	67,7				
**calculated angle beta (degrees)**							
PBM	INTACT	TRANSECTED	TREATED				
10%	51,6	88,9	46,8				
20%	69,0	99,4	44,9				
30%	50,7	97,0	47,9				

Table S27. Legend for abbreverations used in Tables S1-S26

LEGEND	
BM	bodymass
PTL	patellar tendon load
PBM	percentage of bodymass
INTACT	Intact cranial cruciate ligament
TRANSECTED	Transected cranial cruciate ligament
TREATED	After surgical treatment
FW	distances measured in stifles treated with #2 braided non-absorbable multi-strand polyethylene suture (FiberWire, Arthrex Vet Systems) only
FT	distances measured in stifles treated with the non-absorbable suture anchor (FASTak, Arthrex Vet Systems) preloaded with #2 braided non-absorbable multi-strand polyethylene suture (FiberWire, Arthrex Vet Systems)
WD	distances measured in stifles treated with the polylactide absorbable bone anchor (Weldix, VetWelding AG) preloaded with #2 braided non-absorbable multi-strand polyethylene suture (FiberWire, Arthrex Vet Systems)
